# Identification of *L11L* and *L7L* as virulence-related genes in the African swine fever virus genome

**DOI:** 10.3389/fmicb.2024.1345236

**Published:** 2024-01-24

**Authors:** Jiaqi Fan, Jingyuan Zhang, Fengjie Wang, Faming Miao, Han Zhang, Yiqian Jiang, Yu Qi, Yanyan Zhang, Lili Hui, Dan Zhang, Huixian Yue, Xintao Zhou, Qixuan Li, Yu Wang, Teng Chen, Rongliang Hu

**Affiliations:** ^1^College of Life Sciences, Ningxia University, Yinchuan, Ningxia, China; ^2^Key Laboratory of Prevention and Control for African Swine Fever and Other Major Pig Diseases, Ministry of Agriculture and Rural Affairs, Changchun, Jilin, China; ^3^Chinese Academy of Agricultural Sciences Changchun Veterinary Research Institute, Changchun, Jilin, China; ^4^Institute of Rare Diseases, West China Hospital of Sichuan University, Chengdu, Sichuan, China

**Keywords:** African swine fever virus, *L7L*, *L11L*, virulence, recombinant virus

## Abstract

**Introduction:**

African swine fever (ASF) is an infectious disease that causes considerable economic losses in pig farming. The agent of this disease, African swine fever virus (ASFV), is a double-stranded DNA virus with a capsid membrane and a genome that is 170-194 kb in length encoding over 150 proteins. In recent years, several live attenuated strains of ASFV have been studied as vaccine candidates, including the SY18ΔL7-11. This strain features deletion of *L7L*, *L8L*, *L9R*, *L10L* and *L11L* genes and was found to exhibit significantly reduced pathogenicity in pigs, suggesting that these five genes play key roles in virulence.

**Methods:**

Here, we constructed and evaluated the virulence of ASFV mutations with SY18ΔL7, SY18ΔL8, SY18ΔL9, SY18ΔL10, and SY18ΔL11L.

**Results:**

Our findings did not reveal any significant differences in replication efficiency between the single-gene deletion strains and the parental strains. Pigs inoculated with SY18ΔL8L, SY18ΔL9R and SY18ΔL10L exhibited clinical signs similar to those inoculated with the parental strains. Survival rate of pigs inoculated with 10^3.0^TCID_50_ of SY18ΔL7L was 25%, while all pigs inoculated with 10^3.0^TCID_50_ of SY18ΔL11L survived, and 50% inoculated with 10^6.0^TCID^50^ SY18ΔL11L survived.

**Discussion:**

The results indicate that *L8L*, *L9R* and *L10L* do not affect ASFV SY18 virulence, while the *L7L* and *L11L* are associated with virulence.

## 1 Introduction

African swine fever virus (ASFV) is the sole member of the *Asfarviridae* family and *Asfivirus* genus (Iyer et al., 2006). ASFV is a double-stranded DNA virus that replicates in the cytoplasm, and has a genome approximately 170–194 kb in length, containing 150–167 open reading frames (ORFs), and encoding over 150 proteins (Chapman et al., 2011). Various genes in ASFV genome cause clinical differences, resulting in chronic, subacute, acute, and hyperacute forms of infection in pigs, with domestic pigs typically exhibiting pronounced clinical signs (Gómez-Villamandos et al., 2013). Pigs with acute ASF infection generally succumb within about 10 days, displaying symptoms like high fever, anorexia, prostration, and cyanosis. In contrast, pigs with hyperacute ASF infection show no symptoms, and die within a week (Bosch-Camós et al., 2020). The mortality rate for subacute and chronic ASF is less than 100%. Subacute and chronic forms of the disease are mainly characterized by less virulent strains with a longer course of disease and Chronic disease signs (include intermittent fever, arthritis, weight loss, and skin ulcers; Sun et al., 2021a).

ASF was first identified in Kenya in 1921 (Wardley et al., 1983). Genotype I ASFV, initially transmitted from Africa in 1957, has been eradicated from all countries and regions except Sardinia, Italy, and Africa, through aggressive prevention and control measures (Mur et al., 2016; Dixon et al., 2019). Since its introduction from Africa to the Republic of Georgia in 2007, genotype II ASFV has continued to spread to neighboring countries (Rowlands et al., 2008). In 2018, ASFV emerged in China and several other Asian countries, and posed a significant threat to the global pig industry (Zhou et al., 2018; Ankhanbaatar et al., 2021; Mai et al., 2021). Endemic in China for nearly 5 years, ASFV was later detected in surveillance studies, which have identified naturally mutated low-virulence genotype II strains in swine farms (Sun et al., 2021b). Additionally, genotype I ASFV has been isolated from farms in China’s Shandong and Henan provinces (Sun et al., 2021a). The emergence of low-virulence and genotype I ASFV presents new challenges in the diagnosis, treatment, prevention, and control of ASF in China (Urbano and Ferreira, 2022).

Researchers worldwide have strived to develop effective vaccines to prevent and control ASFV infections (Borca et al., 2020; Zhang Y. et al., 2021). In these endeavors, live attenuated vaccines (LAVs) have become a significant focus. LAVs are created by deleting one or more specific genes in ASFV through homologous recombination, leading to attenuated virulence and resistance to highly virulent parental strains. Examples include ASFV-G-ΔI177L (Borca et al., 2020), SY18∆I226R (Zhang Y. et al., 2021), ASFV-GZ∆I73R (Liu et al., 2023a), ASFV-G-∆A137R (Gladue et al., 2021), ASFV-ΔQP509L/QP383R (Li et al., 2022), ASFV-G-ΔMGF (Deutschmann et al., 2022), ASFV-G-ΔI177L/ΔLVR (Borca et al., 2021), ASFV SY18∆L7-11 (Zhang J. et al., 2021). ASFV SY18ΔL7–11 is an attenuated strain obtained by deleting the ORFs of the *L7L* (alternative name *I7L*), *L8L* (*I8L*), *L9R* (*I9R*), *L10L* (*I10L*), and *L11L* genes in the highly virulent strain ASFV SY18. The deletion of *L7L-L11L* from ASFV SY18 genome was not found to alter the ability of the virus to replicate *in vitro*. After intramuscular injection of animals with SY18ΔL7–11 at 10^3.0^TCID_50_ and 10^6.0^TCID_50_, all pigs, except for one in the 10^3.0^TCID_50_-inoculated group, died during a 21-day observation period. Thus, L7L-L11L genes were not associated with replication, but instead virulence. Therefore, *L7L*, *L8L*, *L9R*, *L10L* and *L11L* were separately deleted from ASFV SY18 genome here to identify which of these genes were related to virulence. Our results showed that pigs inoculated with the virus at 10^3.0^TCID_50_ had overall survival rates of 25 and 100% upon deletion of *L7L* and *L11L*, respectively. The overall survival rate was 0% in the remaining three groups. Thus, deletion of the *L7L* and *L11L* genes reduced the virulence of the virus.

## 2 Materials and methods

### 2.1 Cells and viruses

Porcine primary alveolar macrophages (PAMs) were prepared as described previously (Zhang Y. et al., 2021). The viral titer assay was performed according to the method described by Reed and Muench. The strain ASFV SY18 (GenBank number: MH766894) used is a porcine-derived isolate from the Changchun Institute of Veterinary Medicine, Chinese Academy of Agricultural Sciences (Zhou et al., 2018).

### 2.2 Multiple sequence alignment of L7L-L11 gene sequences

Twelve ASFV strains of seven genotypes were selected to perform a multiple sequence alignment of *L7L*, *L8L*, *L9R*, *L10L*, and *L11L* fragments. For this purpose, the MAFFT website was used, and the Jalview was used to visualize alignment results.

### 2.3 Construction of ASFV mutants

The recombinant transfer vector (p72EGFPΔL7L) contained a fragment of about 1,200 bp of the gene flanking the L7L gene, and the p72 promoter EGFP gene cassette. p72EGFPΔL8L, p72EGFPΔL9R, p72EGFPΔL10L, and p72EGFPΔL11L were obtained identically. PAM cells (2 × 10^6^) PAM cells were spread into a 6-well plate, the recombinant transfer vector was added to the cells using Jet-Macrophage (Polyplus) transfection reagent, 1 MOI of ASFV SY18 was added after 4 h, and fluorescence was observed after 24 h. The fluorescence intensity was screened, and the purified virus was obtained using the limited dilution method. Polymerase chain reaction was used to identify the ASFV mutants, and specific sequences are shown in Table 1. Recombinant viral DNA was extracted and sent to Novogene (Tianjin, China) for next-generation sequencing.

**Table 1:** Primer information.

Virus	Forward Primer (5′-3′)	Reverse Primer (5′-3′)
SY18△L7L, SY18△L8L	TGGTAGTATTGTCCAAACCG	TAGGGACTTATGTAGTTTCGTC
SY18△L9R, SY18△L10L	TCTTATGGATGGACGACCTC	GGATTTGGACGACTTGGTC
SY18△L11L	ACATATGATGTCTAGGAATA	AACTTATAACAATTGCACTC

### 2.4 Viral growth curves

A total of 2 × 106 PAM cells were spread into 6-well plates, and infected with ASFV SY18, SY18ΔL7L, SY18ΔL8L, SY18ΔL9R, SY18ΔL10L, SY18ΔL11L at an amount of 0.1 MOI. The samples were collected at 2, 12, 24, 48, 72, and 96 h after infection. The samples were then freeze-thawed three times, and the TCID50 of each sample was determined.

### 2.5 Animal experiments

Experiments were conducted according to standard procedures approved by the Animal Welfare and Ethics Committee of the Changchun Veterinary Research Institute and the Animal Biosafety Level 3 (ABSL-3) Laboratory.

#### 2.5.1 Experiment 1

The virulences of SY18ΔL7L, SY18ΔL8L, SY18ΔL9R, SY18ΔL10L, SY18ΔL11L with respect to ASFV SY18 were assessed using commercial pigs weighing around 15 kg. Each group of pigs (*n* = 4) was inoculated intramuscularly (i.m.) with 10^3.0^TCID_50_ of the virus. Clinical symptoms and temperature changes were recorded daily throughout the experiment, and blood was collected on days 0, 3, 7, 10, 14, and 21 for nucleic acid and antibody detection. The experimental observation period was 21 days, and surviving animals were euthanized. Viral loads in the tissues and organs of each test animal were determined.

#### 2.5.2 Experiment 2

Using commercial pigs around 15 kg, each group of pigs (n = 4) was injected intramuscularly with 10^3.0^TCID_50_ SY18ΔL11L, 10^6.0^TCID_50_ SY18ΔL11L and an equal amount of saline as a negative control. After 28 days after inoculation, each group of animals was inoculated intramuscularly with 10^3.0^TCID_50_ ASFV SY18. Clinical performance and temperature changes were detected daily during the test period, and blood was collected every 7 days for nucleic acid and antibody detection. At the end of the test, the surviving animals were euthanized and the viral load in the tissues and organs of the test animals was detected.

### 2.6 Anti-African swine fever p54 antibody assay

Indirect ELISA was used to detect anti-p54 antibodies. Anti-p54 antibodies were detected in each serum sample as previously described, and sample optical density (OD) values were measured at 450 nm. Samples were considered antibody positive when the sample OD450 nm/positive control OD450 nm (S/P) was greater than 0.25.

## 3 Results

### 3.1 Genetic diversity of L7L-L11L of ASFV strains

**Figure 1:**
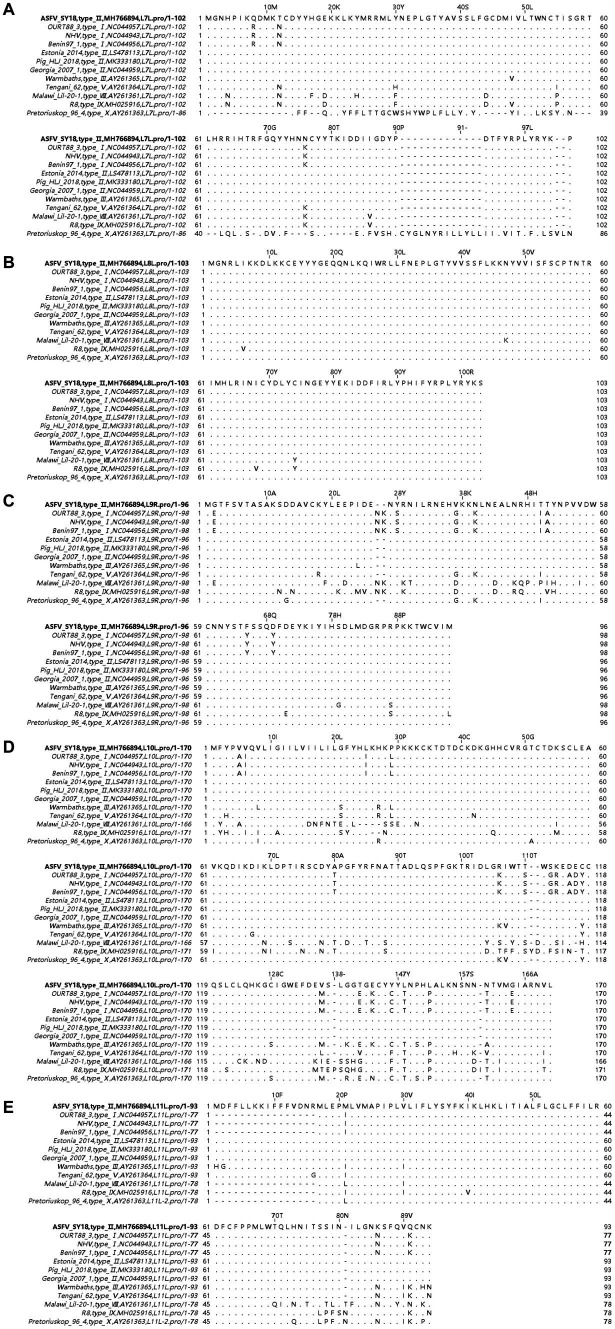
Multiple sequence alignment of amino acid sequences of *L7L-L11L*. Panels **(A–E)** show sequences of *L7L, L8L, L9R, L10L*, and *L11L*, respectively. The sequence of ASFV SY18 is placed on the top row for reference. Amino acids are indicated by their respective single letter codes. Matches are indicated with “. “, and gaps are denoted with “-.” Sequences were aligned using the MAFFT algorithm and Jalview software (Accessed 5 October 2023).

**Figure 2:**
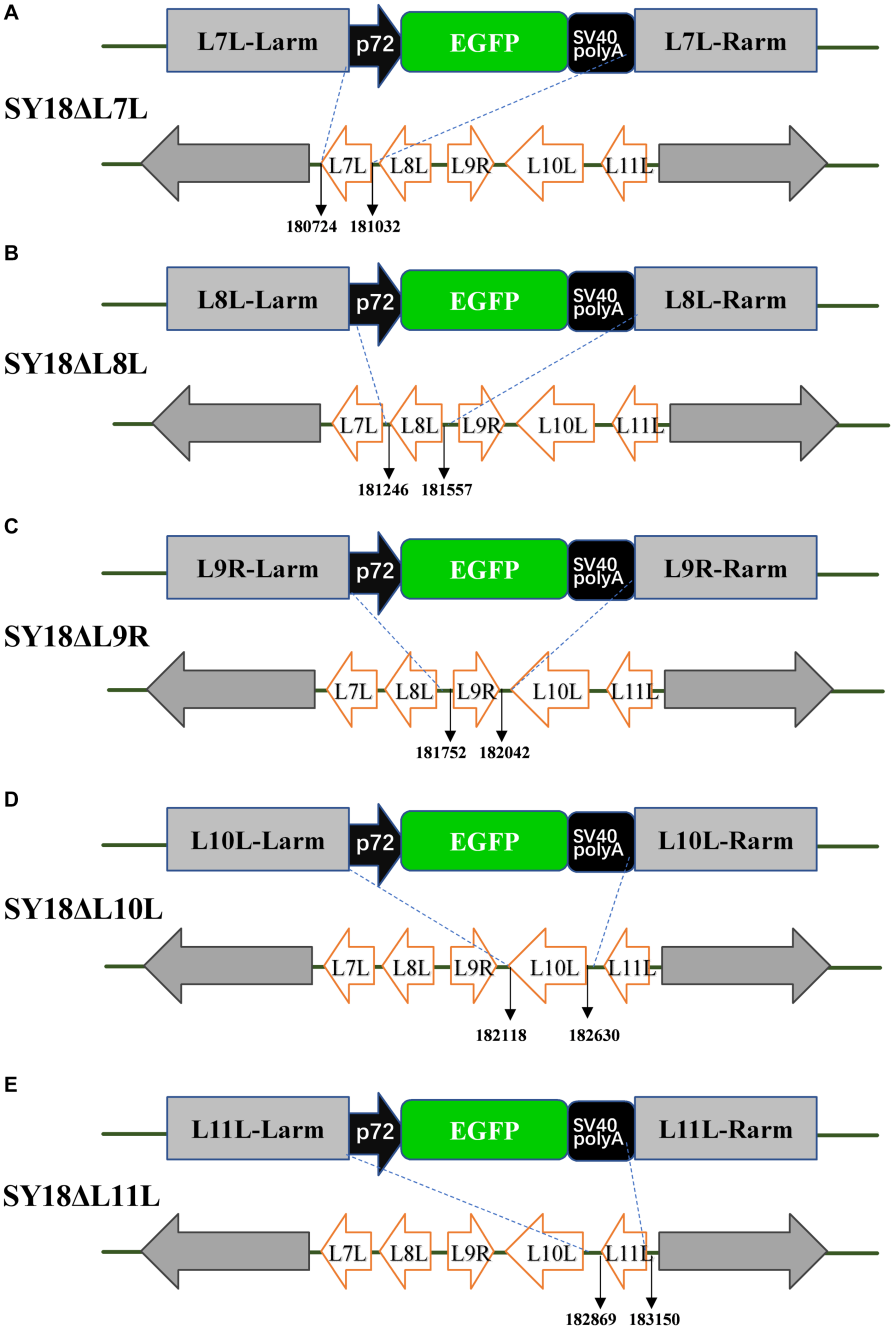
Schematic diagrams of recombinant viral constructs. **(A)** A schematic diagram of the SY18ΔL7L construct ASFV SY18 in which the *L7L* was replaced by a green fluorescent protein expression cassette. Panels **(B–E)** indicate the schematic diagrams of the constructs of SY18ΔL8L, SY18ΔL9R, SY18ΔL10L, and SY18ΔL11L, respectively, where the target genes were replaced by an EGFP expression cassette.

A total of 309 bp in *L7L* encodes 102-amino-acids, 312 bp in *L8L* encodes 103, 291 bp in *L9R* encodes 96, 531 bp in *L10L* encodes 170, and 282 bp in *L11L* encodes 93 (Figure 1). They were located between nucleotide positions 180,724 and 181,032 of the reverse strand, 181,246 and 181,557 of the antisense strand, 181,752 and 182,042 of the plus strand, 182,118 and 182,630 of the antisense strand, and 182,869 and 183,150 of the antisense strand of the ASFV SY18 genome (Figure 2). Degree of conservation of *L7L, L8L, L9R, L10L*, and *L11L* in ASFV isolates of multiple genotypes. A comparison of the amino acid sequences of the isolate ASFV SY18 with those of genotype II strains isolated from Heilongjiang, China (Pig/HLJ/2018), Georgia (Georgia/2007/1), and Estonia (Estonia/2014) revealed that the amino acid sequences of *L7L-L11L* were completely identical. The amino acid sequence of *L8L* was identical in genotype I and II isolates. The amino acid sequences of *L7L, L8L, L9R, L10L*, and *L11L* were identical in the same genotype, and a large gap was observed between the different genotypes. The *L7L* sequence in the Pretoriuskop/96/4 strain (genotype X) was significantly different than those in other strains. The ASFV genotype II is prevalent globally. Positions 1, 17, 21, 24, and 67 of *L11L* in genotype II ASFV SY18, Pig/HLJ/2018, Georgia/2007/1, and Estonia/2014 included a methionine. However, *L11L* of OURT88/3, NHV, Benin97/1 (genotype I) had 77 amino acids, *L11L* of Malawi Lil/20/1 (genotype VIII) has 78, *L11L* of R8 (genotype IX) had 78, *L11L* of Pretoriuskop/96/4 (genotype XX) had 78 amino acids. These strains did not include the first 16 amino acids found in *L11L* of ASFV SY18, with the 17th amino acid in the L11L of ASFV SY18 being the most common initial amino acid (the second occurrence of methionine in the *L11L* of genotype II). The *L11L* of Tengani/62 (genotype V) contained 93 amino acids. The *L11L* of warm baths (genotype III) had the same sequence of amino acids between positions 3 and16 compared to *L11L* of ASFV SY18, except that the first amino acid was not methionine. Therefore, the first 16 amino acids of *L11L* in warm baths were also non-functional.

### 3.2 Creation of ASFV mutants

**Figure 3:**
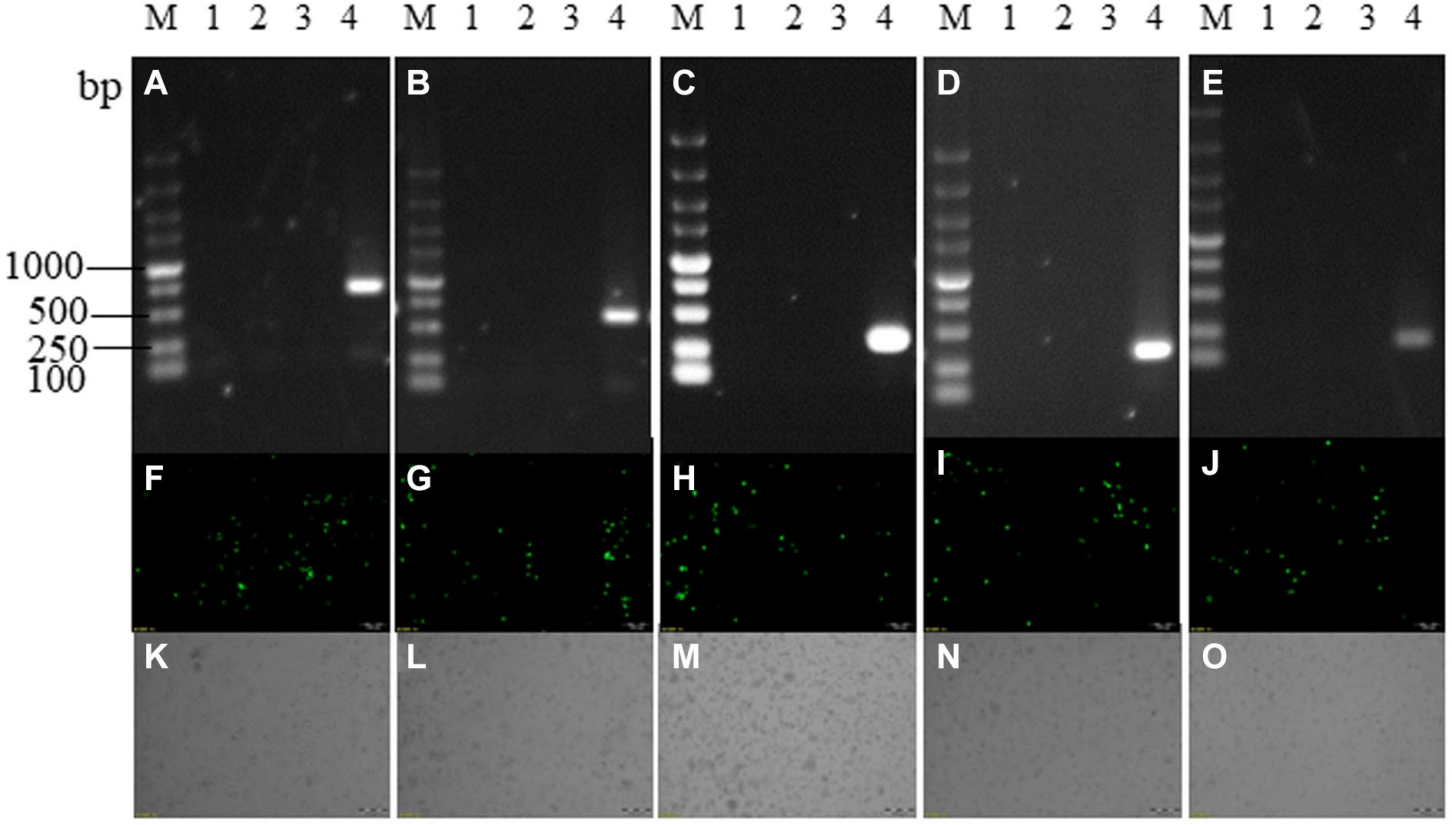
Recombinant virus identification. Panels **(A–E)** are PCR identification and purification results of SY18ΔL7L, SY18ΔL8L, SY18ΔL9R, SY18ΔL10L, SY18ΔL11L, panels **(F–J)** are the fluorescence maps of each single-gene deletion viruses, and panels **(K–O)** are the bright field of each single-gene deletion viruses. M: DL5000 marker; a1~a3, b1~b3, c1~c3, d1~d3, e1~e3: each single-gene deletion virus detection samples; 4: ASFV SY18 control.SY18ΔL7L and SY18ΔL8L amplified a 643 bp fragment in the presence of ASFV SY18; SY18ΔL9R amplified a 280 bp fragment in the presence of ASFV SY18; SY18ΔL10L, and SY18ΔL11L amplified a 200 bp fragment in the presence of ASFV SY18.

In order to identify the specific virulence genes among *L7L-L11L*, we designed and constructed single-gene deletion strains (SY18ΔL7L, SY18ΔL8L, SY18ΔL9R, SY18ΔL10L, and SY18ΔL11L). The construction method is shown in Figure 2. Target genes were replaced with a green fluorescent gene cassette containing the p72 promoter using a recombinant method. The recombinant viruses were purified using fluorescence activity. The PCR assay determined that the target bands of the gene were not detected, whereas the ASFV SY18 control showed the target bands, confirming that single-gene deletion viruses missing each target gene were successfully obtained (Figure 3). Comparison of the whole-genome sequences of the ASFV mutant and parental ASFV SY18 using second-generation sequencing revealed that, apart from design changes, no unwanted mutations were detected in the genomes of the constructed mutants.

### 3.3 Replication of ASFV mutants in porcine macrophages

**Figure 4:**
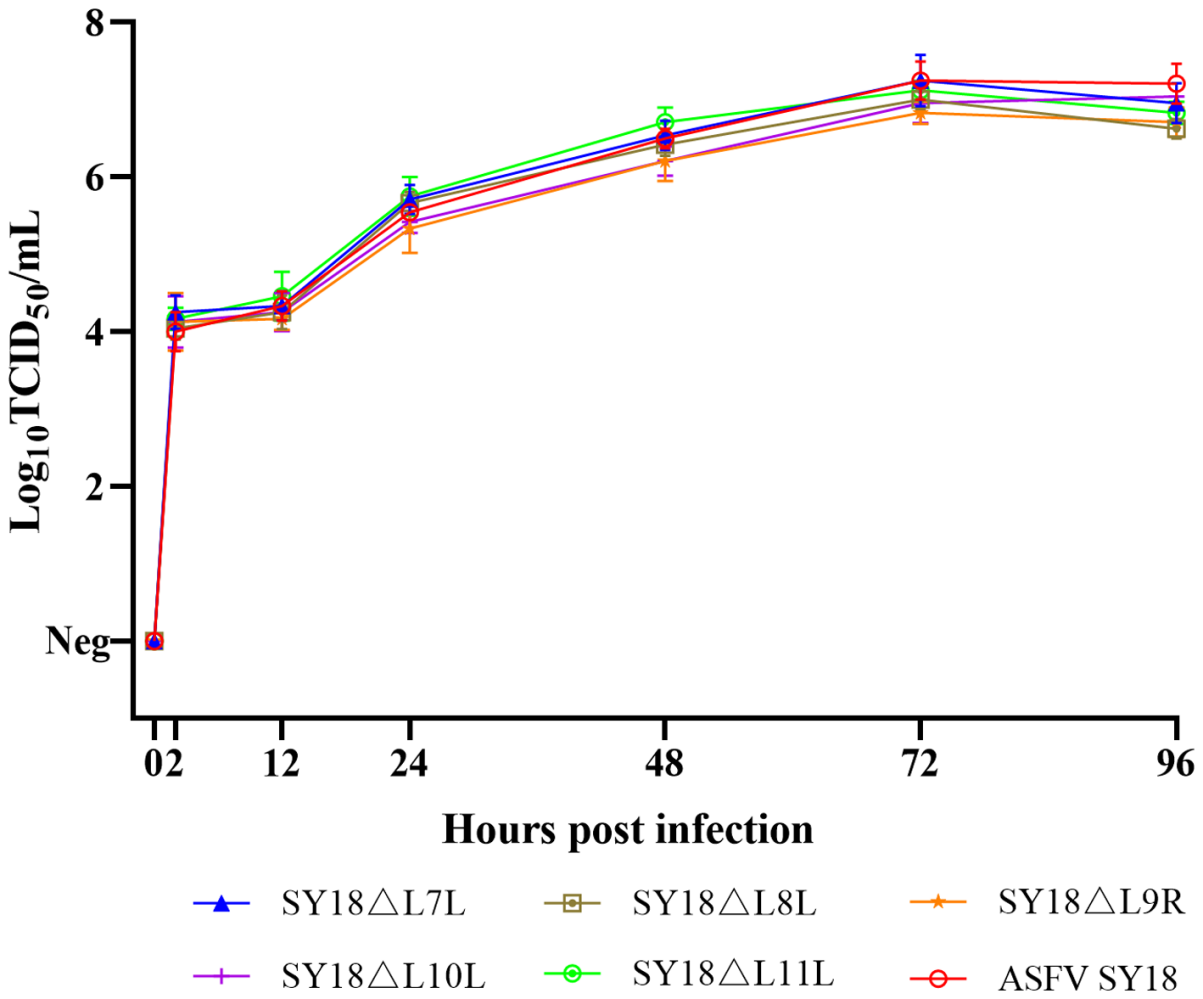
*In vitro* growth characteristics of recombinant viruses. PAM was infected with ASFV SY18, SY18ΔL7L, SY18ΔL8L, SY18ΔL9R, SY18ΔL10L, SY18ΔL11L at 0.1 MOI, and the viral titers of three independent experimental samples were determined at different times of infection. y-axis represents the viral titer expressed as log10 TCID_50_/mL, and the x-axis represents the time after infection (hours).

Macrophages were infected with each deletion strain at an MOI of 0.1, and the parental ASFV SY18 was used as a control. Samples were collected at 2, 12, 24, 48, 72, and 96 h post-infection (hpi). The results shown in Figure 4 revealed no significant differences between the growth curves of single-gene deletion viruses in target cells than those of the parental strains *in vitro*. There was also no difference in the replication pattern between the single-gene deletion strains, all of which reached the highest titer at 72 hpi. These findings demonstrate that the deletion of *L7L*, *L8L*, *L9R*, *L10L*, and *L11L* did not affect the proliferative ability of ASFV in macrophages *in vitro*.

### 3.4 Evaluation of the virulence of ASFV mutants

**Figure 5:**
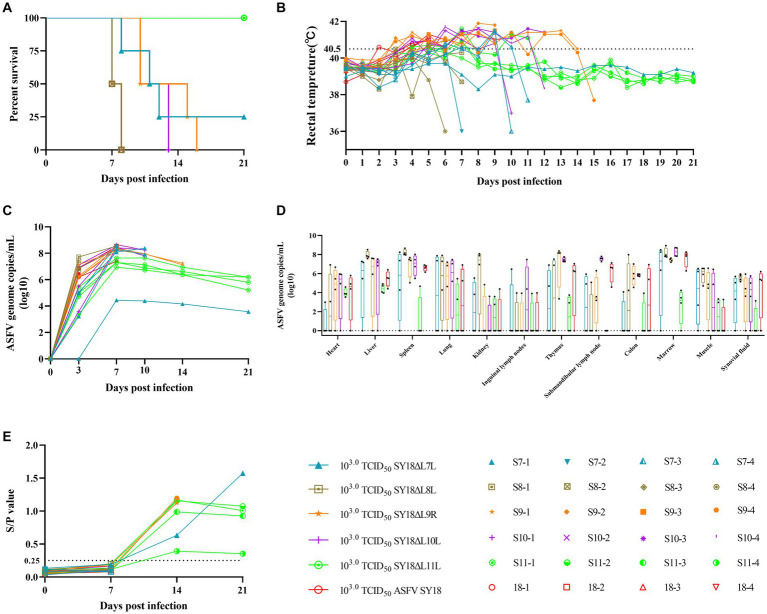
Survival, rectal temperature, viraemia, viral load in tissues and anti-p54 antibody levels in pigs inoculated with 10^3.0^ TCID_50_ ASFV recombinant virus. **(A)** Survival of animals in each group. **(B)** Each line represents temperature data for individual animals. The dashed line indicates the fever threshold of 40.5°C. **(C)** Detection of ASFV genomic DNA in porcine blood. **(D)** Detection of ASFV genomic DNA in porcine tissues. **(E)** Anti-p54 antibody level in pig serum. y-axis represents the S/P ratio (sample OD_450nm_/positive control OD_450nm_), x-axis represents the number of days of immunization, and the dotted line represents the critical value of 0.25.

To assess the virulence of SY18ΔL7L, SY18ΔL8L, SY18ΔL9R, SY18ΔL10L, SY18ΔL11L, and ASFV SY18 (positive control group) for pigs, each group was injected intramuscularly with 10^3.0^ TCID_50_ of virus. The survival and body temperature results are shown in Figures 5A,B and Table 2. Pigs in the ASFV SY18 group became febrile on day 4 after infection, and showed typical symptoms of ASF, and died on day 8 post infection. Pigs in the SY18ΔL7L group showed persistent fever between days 1 and 5. Three of these pigs died on the 9th, 12th, and 13th dpi, and one pig (S7-1) survived during the observation period. Pigs in the SY18ΔL8L group died on days 7 and 8 post-infection, which was consistent with the timing of death in the control group. However, only one animal in the SY18ΔL8L group showed elevated body temperature, whereas the remaining three animals had normal body temperatures. Pigs in the SY18ΔL9R group died 10 to 16 days and pigs in the SY18ΔL10L group died 10 to 13 days post infection, respectively. Deaths in both groups delayed backwards compared with the control. All pigs in the SY18ΔL11L group survived the 21 days of observation, with three pigs experiencing a transient increase in body temperature and one returning to normal temperature after 6 days of fever.

**Table 2:** Clinical manifestations and survival after immunization with ASFV mutants.

Groups	No. of survivors/total	No. of fever/total	Mean value for fever parameter (SD) Fever (≥40.5°C)		
			Day of onset (days ± SD)	Duration no. of days (days ± SD)	Highest rectal temp (°C ± SD)
10^3.0^ TCID_50_ SY18ΔL7L	1/4	3/4	6.67 ± 0.47	2.00 ± 1.58	40.80 ± 0.72
10^3.0^ TCID_50_ SY18ΔL8L	0/4	1/4	5^a^	2^a^	40.25 ± 0.28
10^3.0^ TCID_50_ SY18ΔL9R	0/4	4/4	4.45 ± 0.83	7.75 ± 3.11	41.56 ± 0.19
10^3.0^ TCID_50_ SY18ΔL10L	0/4	4/4	4.50 ± 0.50	6.50 ± 1.11	41.60 ± 0.07
10^3.0^ TCID_50_ SY18ΔL11L	4/4	4/4	5.75 ± 1.30	2.25 ± 2.16	40.85 ± 0.22
10^3.0^ TCID_50_ ASFV SY18	0/4	4/4	4.00 ± 0.00	2.50 ± 0.50	41.03 ± 0.04

^a^Only 1 pig in this group had fever and the rest of the pigs did not die directly after the fever was detected.

The viral loads in the blood of the experimental animals are shown in Figure 5C. Viral nucleic acids were detected in the blood of all animals on day 3 after infection, except for one pig in the SY18ΔL7L group. On the 3rd day of infection, all pigs in the ASFV SY18 group showed high viral loads in their blood (10^6.19^–10^6.86^ copies/mL), and pigs in the SY18ΔL8L and SY18ΔL9R groups similarly showed high viremia (10^6.72^–10^7.72^ copies/mL and 10^6.16^–10^7.13^ copies/mL). On the 3rd day of infection, pigs in the SY18ΔL7L, SY18ΔL10L, and SY18ΔL11L groups had lower viral loads in their blood compared to the control group (0–10^5.08^ copies/mL, 10^3.58^–10^7.12^ copies/mL, and 10^3.37^–10^5.42^ copies/mL, respectively). The highest viral levels in the blood of all animals were reached on the 7th dpi. Subsequently, the viral load in the blood of surviving animals tended to decrease. The presence of virus was not detected in the blood of 1 pig without fever (S7-1) in the SY18ΔL7L group on the 3rd day, and the presence of the virus was detected on the 7th day, after which and the virus level was consistently low (10^3.56^–10^4.44^ copies/mL).

### 3.5 Viral load of pig tissues after infection with ASFV mutants

The pigs used in Experiment 1 were dissected, and organs including the heart, liver, spleen, lungs, kidneys, inguinal lymph nodes, thymus, submandibular lymph nodes, colon, bone marrow, muscles, and joint fluids were collected and tested for the presence of the virus. Figure 5D indicates that most animals had the virus in their organs, with the spleen, liver, and bone marrow showing the highest detection rates. In the SY18ΔL11L group, both the detection rate and viral load in the organs were lower compared to other groups. In the SY18ΔL7L group of non-febrile pigs (S7-1), post-dissection testing of tissues showed no viral nucleic acid except in the muscle tissue (10^2.68^ copies/mL).

Specific antibody responses in pigs infected with ASFV mutants. To assess the specific immune response, we determined the levels of anti-p54 antibodies in the serum. As shown in Figure 5E, the sera of each animal were negative for anti-p54 antibodies on day 7 of immunization, and those that survived until day 14 (all pigs in the S9-1, S9-4, S7-1 and SY18ΔL11L groups) were positive for anti-p54 antibodies. Pigs S9-1 and S9-4 produced higher levels of anti-p54 antibodies (S/*p* values of 1.123 and 1.197, respectively), yet died on days 15th and 16th days. The S11-4 pigs in the SY18ΔL11L group expressed lower anti-p54 antibodies than S9-1, S9-4, but the S11-4 pigs survived the observation period. This finding demonstrates that the expression of ASF-specific antibodies is not a marker for animal survival.

### 3.6 Assessment of protective capacity of SY18ΔL11L mutant

**Figure 6:**
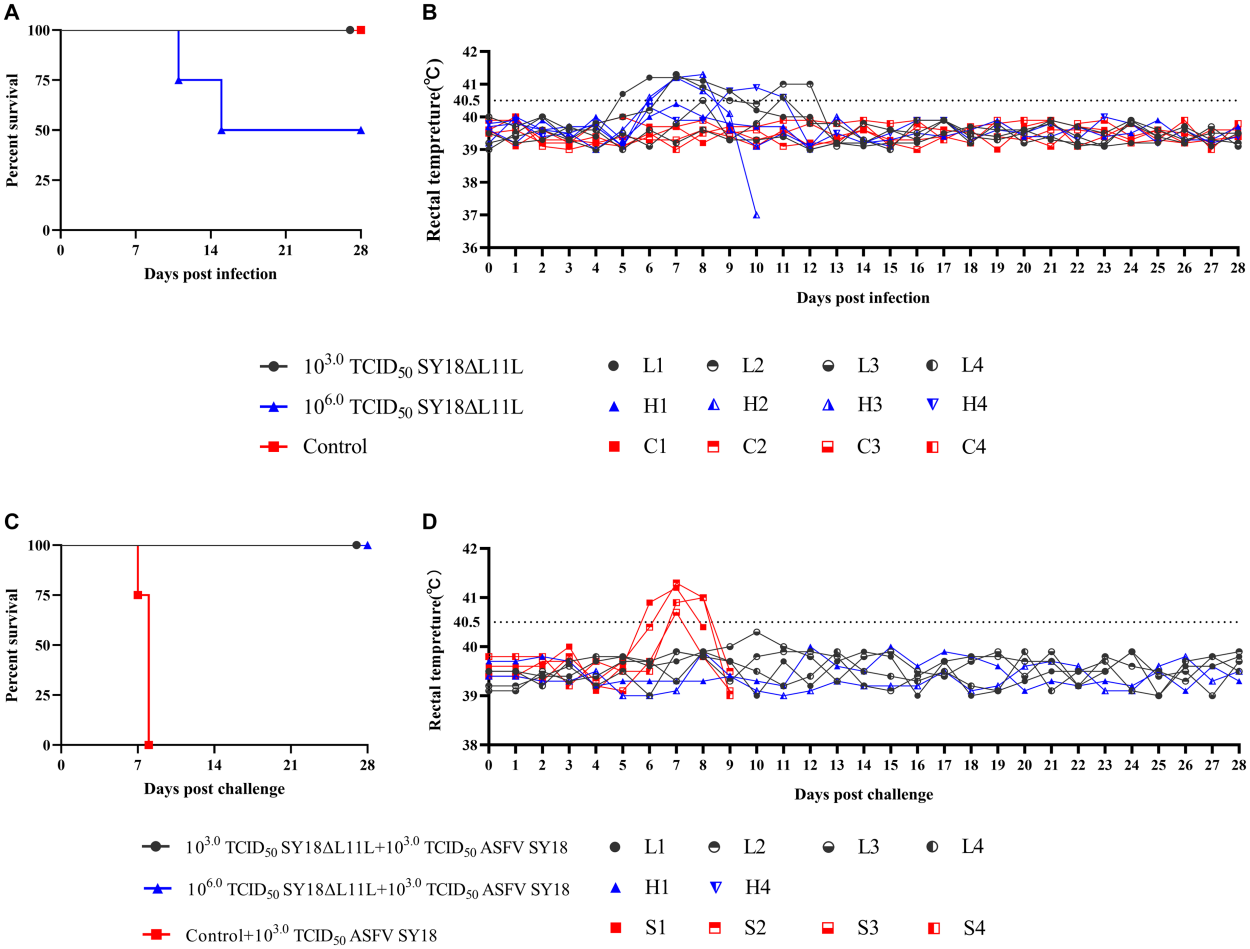
Survival and body temperature of pigs after immunization and attack challenge. Survival **(A)** and rectal temperature **(B)** of pigs immunized with 10^3.0^ TCID_50_ SY18ΔL11L, 10^6.0^ TCID_50_ SY18ΔL11L and control saline. Survival **(C)** and rectal temperature **(D)** of pigs challenged with 10^3.0^ TCID_50_ ASFV SY18 tapping. Each line in the graph represents body temperature data for a single animal.

To assess the degree of attenuation of SY18ΔL11L, a low dose (10^3.0^TCID_50_) and a high dose (10^6.0^TCID_50_) of SY18ΔL11L were inoculated intramuscularly. Another group of pigs was injected with an equal amount of saline as control (Figures 6A,B; Table 3). All pigs in the low-dose SY18ΔL11L-infected group survived, with two pigs experiencing a transient increase in body temperature and two pigs having persistent fever for 4–5 days. All animals in the high-dose SY18ΔL11L-infected group showed elevated body temperatures (>40.5°C), with symptoms of ASF in H-2 and H-3, which were euthanized on days 11th and 15th days post-inoculated due to the severity of the disease in both animals. Pigs in the control group showed no adverse reactions. To determine the response of the surviving pigs when challenged with the parental virus, a virus provocation test was performed on all pigs that survived the inoculation observation period (Figures 6C,D; Table 4). Each pig was intramuscularly injected with 10^3.0^TCID_50_ of SY18 cells, and observed for 28 days. During the viral challenge, all pigs in the control group had elevated temperatures, developed an ASF clinical reaction, and were euthanized by the 9th day post-challenge (dpc). Pigs inoculated with SY18ΔL11L all survived during the challenge.

**Table 3:** Clinical performance and survival of pigs immunized with SY18△L11L.

Groups	No. of survivors/total	No. of fever/total	Mean value for fever parameter (SD) Fever (≥40.5°C)		
			Day of onset (days ± SD)	Duration no. of days (days ± SD)	Highest rectal temp (°C ± SD)
10^3.0^ TCID_50_ SY18ΔL11L	4/4	4/4	7.75 ± 2.17	3 ± 2	40.9 ± 0.35
10^6.0^ TCID_50_ SY18ΔL11L	2/4	4/4	7 ± 1.22	2.75 ± 1.09	40.9 ± 0.31
Mock	4/4	0/4	a	a	a

^a^The pigs did not have a fever.

**Table 4:** Clinical performance and survival rate after challenge.

Groups	No. of survivors/total	No. of fever/total	Mean value for fever parameter (SD) Fever (≥40.5°C)		
			Day of onset (days ± SD)	Duration no. of days (days ± SD)	Highest rectal temp (°C ± SD)
10^3.0^ TCID_50_ SY18ΔL11L + 10^3.0^ TCID_50_ ASFV SY18	4/4	0/4	a	a	a
10^6.0^ TCID_50_ SY18ΔL11L + 10^3.0^ TCID_50_ ASFV SY18	2/2	0/2	a	a	a
10^3.0^ TCID_50_ ASFV SY18	0/4	3/4	5.57 ± 0.47	2.33 ± 0.47	41.17 ± 0.12

^a^The pigs did not have a fever.

**Figure 7:**
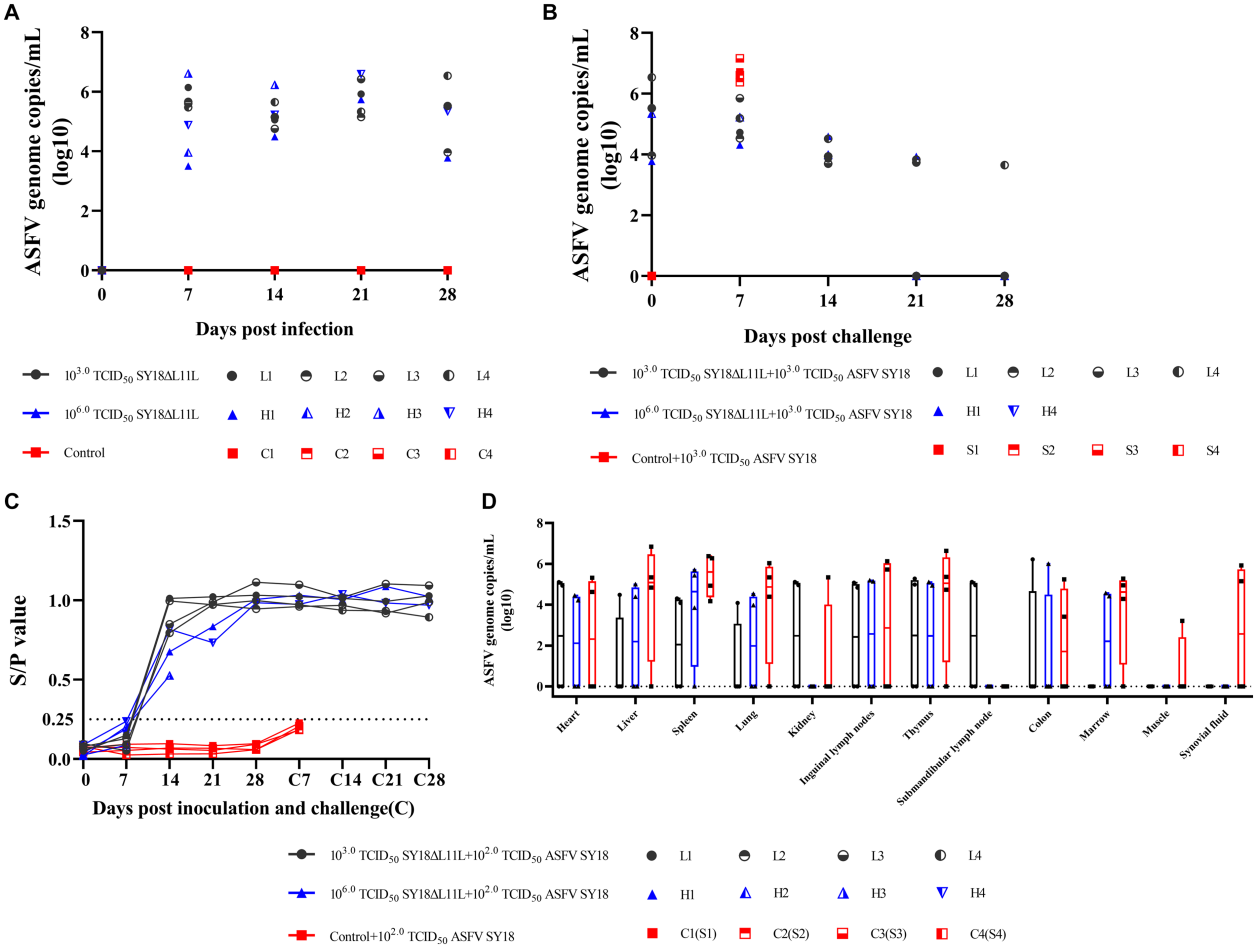
Viraemia, changes in antibody levels and tissue viral content in pigs after immunization and attack attacks. Detection of ASFV genomic DNA in the blood of pigs after immunization **(A)** and attack challenge **(B)**. y values denote log10 copies/mL and x values denote time of inoculation and challenge. **(C)** Viral loads in tissues of immunized pigs after takedown challenge. Each symbol represents each pig. Values represent log10 copies/mL. H2 and H3 died during immunization and were not challenged with the parental virus; H2 and H3 only represent viral loads in tissues of immunized pigs. **(D)** Anti-p54 antibodies in pig sera after immunization and challenge. y-axis represents the S/P (sample OD_450nm_/positive control OD_450nm_) ratio and x-axis represents the number of days of immunization. The threshold of 0.25 is indicated by the dashed line.

Animals inoculated with SY18ΔL11L strain developed viremia on day 7 after immunization (Figure 7A), with the viral load in blood reaching its maximum on day 7 or 14 post-infection, followed by a slow decrease. After ASFV SY18 challenge, control animals exhibited high viral loads in their blood. SY18ΔL11L-infected animals challenged with the parental virus exhibited a steady decline in blood viral load after an increased on day 7. Then, no virus in blood of all but 1 pig on the 28th dpc was be detected (Figure 7B). The ASFV load in the tissues and organs of the animals was measured at the end of the observation period. The results showed that pigs in the control group had higher viral detection in tissues and higher viral loads (Figure 7C). Pigs that died during the immunization period (H2 and H3) had relatively higher virus detection in tissues compared to other pigs immunized with SY18ΔL11L.

The host antibody responses in animals inoculated with SY18ΔL11L are shown in Figure 7D. Animals inoculated with SY18ΔL11L were weakly positive for anti-p54 antibodies in sera of L3 and H4 on day 7 of immunization, and positive for antibodies on day 14, followed by high levels of antibodies in sera thereafter. H3 pigs in the high-dose SY18ΔL11L group died on day 15 even though they were positive for antibodies in serum on day 14 of immunization. After the takedown challenge, pigs in the control group remained negative, and antibodies in pigs inoculated with SY18ΔL11L remained at high levels.

## 4 Discussion

African swine fever continues to adversely impact the global pig farming industry, and is expanding into new endemic areas. Vaccination remains one of the most effective methods to prevent the spread of this virus. However, the effectiveness of inactivated vaccines has been unsatisfactory (Blome et al., 2014; Cadenas-Fernández et al., 2021). Subunit vaccines have seen limited research due insufficient knowledge on viral proteins (Liu et al., 2023). Effective subunit vaccines are still being investigated (Goatley et al., 2020). Live attenuated virus vaccines, which are produced by passaging tissues or cell cultures to generate ASFV and genetically-engineered and attenuated ASFV, induce strong immunity against ASFV-related strains. After years of ASF epidemics in some areas, a decrease in pig mortality has been observed, leading to isolation of naturally attenuated ASFV that cause subacute, chronic, or subclinical forms of the disease (Arias et al., 2017). However, these have very poor safety profiles, and are prone to causing persistent viremia in pigs, along with symptoms of chronic ASF infection (Gallardo et al., 2018). When ASFV grows in maladapted cells, significant portions of the virus can be lost or mutated, resulting in cell-adapted attenuated ASFV (Koltsova et al., 2021). The number of passages of cell-adapted attenuated ASFV is unpredictable, and immunogenicity decreases when the number of passages is excessive (Krug et al., 2015). Currently, genetically engineered live ASFV with reduced virulence is the most promising ASF vaccine; however, it may not completely clear the virus from pigs, and there is a risk of virulence reversal. Therefore, the construction of ASFV mutants by genetic engineering and the selection of rational virulence-related genes are essential for the development of future ASF vaccines.

ASFV has a genome 170–194 kb in size, containing more than 150 open reading frames (ORFs). Functions of only a small fraction of these ORFs have been characterized. The *L7L-L11L* ORF is located in the right variable region of the ASFV genome, where *L10L* is homologous to *KP177R* (encoding the p22 protein), and *L7L* and L8L genes are thought to be members of MGF110 (Vydelingum et al., 1993; Zhang J. et al., 2021; Cackett et al., 2022). We found that the replication efficiency of the 5 ASFV-mutants was similar to that of the parental strains during *in vitro* experiments. Therefore, *L7L-L11L* are not replication-associated genes. Current research on live attenuated ASFV vaccines has focused on the rational attenuation of virulent viruses through deletion of immunosuppressive genes (Liu et al., 2023b). In a previous study, deletion of the *L7L-L11L* ORF in the ASFV SY18 strain (genotype II) was found to significantly attenuate virulence (Zhang J. et al., 2021). Therefore, virulence-related genes are found in the ORF of *L7L-L11L*. Here, we found that inoculation of pigs with deletion strains of the *L8L*, *L9R*, and *L10L* led to deaths of all the pigs in each group. However, compared with the parental strains, the death time of pigs infected with the deletion strains of the *L9R* and *L10L* genes was delayed to a certain extent, one pig survived after being infected by the deletion strain of the *L7L* gene, and all pigs infected with the deletion strain of the L11L gene survived during the observation period, despite showing fever. We reason that the reduced pathogenicity of SY18ΔL7–11 is the result of the combined deletion of the *L7L* and *L11L* genes.

The virulence of ASFV SY18 was not significantly altered by deletion of *L8L*, and deletion of the *L11L* significantly reduced the pathogenicity of ASFV SY18. Vuono et al. performed a deletion of the *I8L* (L8L) alone in the Georgia2010 strain (genotype II), and reported no effects on viral replication or virulence, which is in agreement with our findings (Vuono et al., 2021). Kleiboeker et al. deleted *L11L* from Malawi Lil-20/1 strain, which resulted in the death of all three pigs inoculated with 10^2.0^HAD_50_, and the *L11L* deletion did not reduce the virulence of Malawi Lil-20/1 (Kleiboeker et al., 1998). Malawi Lil-20/1 is a highly virulent strain isolated in Malawi, Africa, whose p72 type belongs to genotype VIII, whereas the epidemic strain in China is genotype II. The deletion of the same gene in different ASFVs may thus result in different virulence-reducing effects. The *CD2v* gene affects the virulence of BA71, ASFV-Kenya-IX-1033, and ASFV HLJ/18, yet has no effect on the virulence of Lil-20/1 in Malawi (Borca et al., 1998; Hemmink et al., 2022). Deletion of the *DP148R* reduced the virulence of Benin 97/1 but had no effect on the virulence of ASFV Georgia 2007/1 and ASFV HLJ18 (Reis et al., 2017; Chen et al., 2020; Rathakrishnan et al., 2021). This phenomenon also suggests that studies on ASFV genes should take the background of the strains and the differences between them into consideration. Because of space constraints we conducted trials using young strains (weaner pigs or grower pigs). The resistance and immunity of young pigs is lower compared to that finisher pigs. ASF causes abortions in sows in late pregnancy (Lohse et al., 2022). We demonstrated the virulence of five ASFV-mutants only in young pigs. However, the specific effects of these ASFV mutants on finishing pigs or gestating sows are uncertain and will require further experimental studies.

## 5 Conclusion

Previous studies have shown that deletion of *L7L-L11L* genes in ASFV SY18 significantly reduces viral virulence, and that virulence-related genes exist among *L7L-L11L*. In this study, we constructed five recombinant viruses with single-gene deletions using homologous recombination technology, 10^3.0^TCID_50_ doses of the recombinant viruses were used to immunize pigs. All pigs in the SY18ΔL8L, SY18ΔL9R, and SY18ΔL10L immunization groups died during the observation period, while one pig survived in the SY18ΔL7L group, and all pigs in the SY18ΔL11L group survived. The results demonstrated that *L8L*, *L9R*, and *L10L* are not ASFV SY18 virulence genes, whereas *L7L* and *L11L* are related virulence. Pigs were immunized with 10^3.0^TCID_50_ or 10^6.0^TCID_50_ doses of recombinant virus SY18ΔL11L, and virus challenge protection experiments were performed. The results showed that SY18ΔL11L could not be completely detoxified by pigs after immunization, and large doses of immunization could still lead to death. However, surviving pigs could resist attack by the parental strain. Here, we found that SY18ΔL11L is an attenuated strain that protects against the parental strain, but not suitable as a vaccine candidate. *L11L* is an ASFV SY18 virulence gene, whereas *L7L* has a slight effect on virus virulence. An in-depth analysis of the functions of this gene may identify new targets for the development of effective vaccines in the future.

## Data availability statement

The datasets presented in this study can be found in online repositories. The names of the repository/repositories and accession number(s) can be found at: (Genbank: OR944087), SY18ΔL8L (Genbank: OR944088), SY18ΔL9R (Genbank: OR944089), SY18ΔL10L (Genbank: OR944090), and SY18ΔL11L (Genbank: OR944091).

## Ethics statement

The animal study was approved by Animal Welfare and Ethics Committee of the Changchun Veterinary Research Institute. The study was conducted in accordance with the local legislation and institutional requirements.

## Author contributions

JF: Methodology, Validation, Writing – original draft. JZ: Validation, Writing – review & editing. FW: Validation, Writing – review & editing. FM: Validation, Writing – review & editing. HZ: Validation, Writing – review & editing. YJ: Validation, Writing – review & editing. YQ: Validation, Writing – review & editing. YZ: Validation, Writing – review & editing. LH: Validation, Writing – review & editing. DZ: Validation, Writing – review & editing. HY: Validation, Writing – review & editing. XZ: Validation, Writing – review & editing. QL: Validation, Writing – review & editing. YW: Validation, Writing – review & editing. TC: Funding acquisition, Writing – review & editing. RH: Funding acquisition, Writing – review & editing.
